# Supplementary material for: Chemical Profile Analysis of Prosopis laevigata Extracts and Their Topical Anti-Inflammatory and Antibacterial Activities
Source: Plants (Basel). 2025 Apr 3;14(7):1118. doi: 10.3390/plants14071118 (PMC11991005; doi:10.3390/plants14071118)
Supplement: Supplementary file 1 [file plants-14-01118-s001.zip › plants-3503242-supplementary.pdf]

Supplementary Data

Chemical Profile Analysis of *Prosopis laevigata* Extracts and Their Topical Anti-Inflammatory and Antibacterial Activities

Manasés González-Cortazar [1,*], David Osvaldo Salinas-Sánchez[2,4,*], Maribel Herrera-Ruiz [1], Paulina Hernández-Hernández [1,4], Alejandro Zamilpa[1], Enrique Jiménez-Ferrer [1], Beatriz Elizabeth Utrera[3], Ma. Dolores Pérez-García[1], Ana S. Gutiérrez-Roman [1,5], Ever A. Ble-González[3].

1 Centro de Investigación Biomédica del Sur, Instituto Mexicano del Seguro Social, Argentina No. 1, Col. Centro, Xochitepec, Morelos 62790, México; cibis_herj@yahoo.com.mx (M.H.-R.); azamilpa_2000@yahoo.com.mx (A.Z.); lola_as@yahoo.com.mx (Ma.D.P.-G.).

2 Centro de Investigación en Biodiversidad y conservación (CIByC), Universidad Autónoma del Estado de Morelos (UAEM). Av. Universidad 1001, Col. Chamilpa, 62209, Cuernavaca Morelos

3 Universidad Juárez Autónoma de Tabasco, carretera Cunduacán-Jalpa Km. 0.5, Cunduacán Tabasco 86690. México; ble_49@hotmail.com (E.A.B.-G.).

4 Escuela de Estudios Superiores del Jicarero (EESJ), Universidad Autónoma del Estado de Morelos, Carretera Galeana-Tequesquitengo s/n Col. el Jicarero 62909. Jojutla, Morelos, Mexico.

5 Centro de Desarrollo de Productos Bióticos, Instituto Politécnico Nacional, Carr Yautepec-Jojutla s/n km 85, San Isidro, 62739, San Isidro, Morelos, Mexico.

* Correspondence: gmanases2000@gmail.com (M. G.-C.) Tel.: +52(777)3612194 and davidos@uaem.mx (D.O.S.-S.) Tel.: +52(777)3297019 ext. 3278

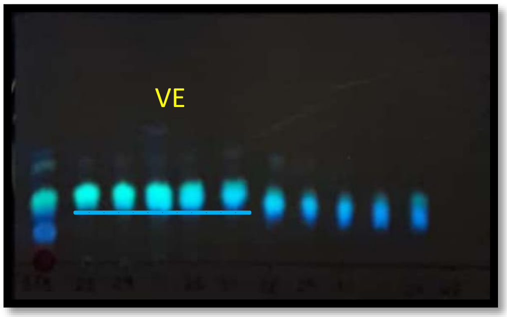

Figure 1S. Thin layer chromatography RP-18 of the isolation of compound VE. Elution systems 50:50 H_2O/Acetonitrile

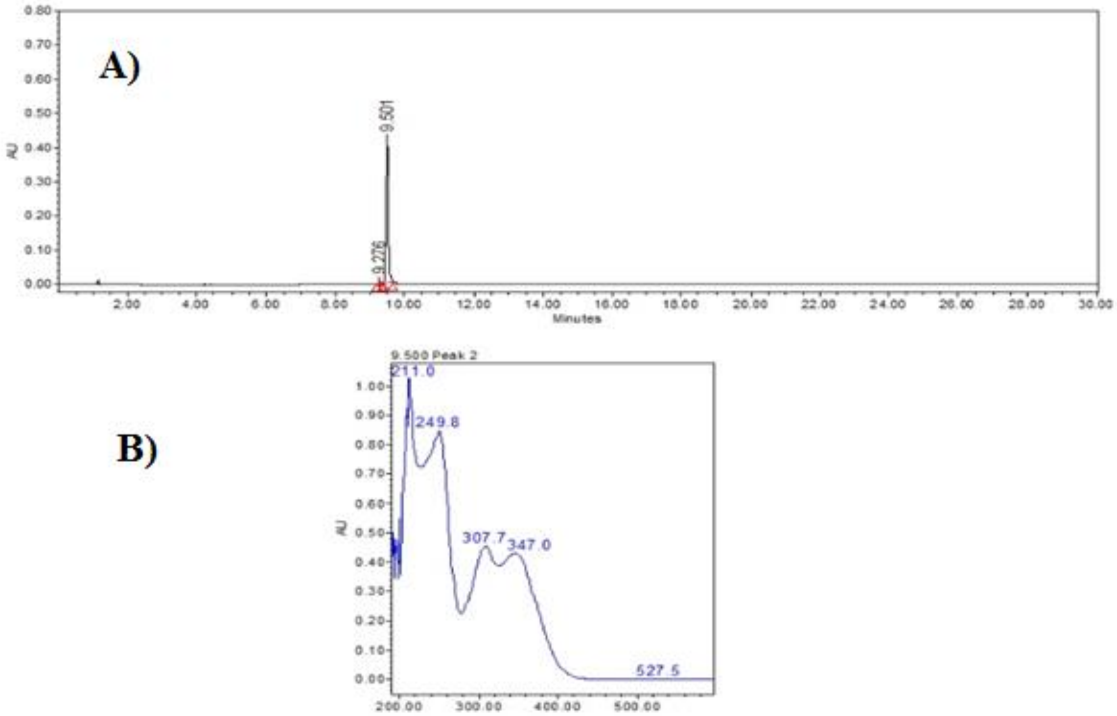

Figure 2S. A) HPLC chromatogram of ethyl veratrate (VE) and B) UV spectrum

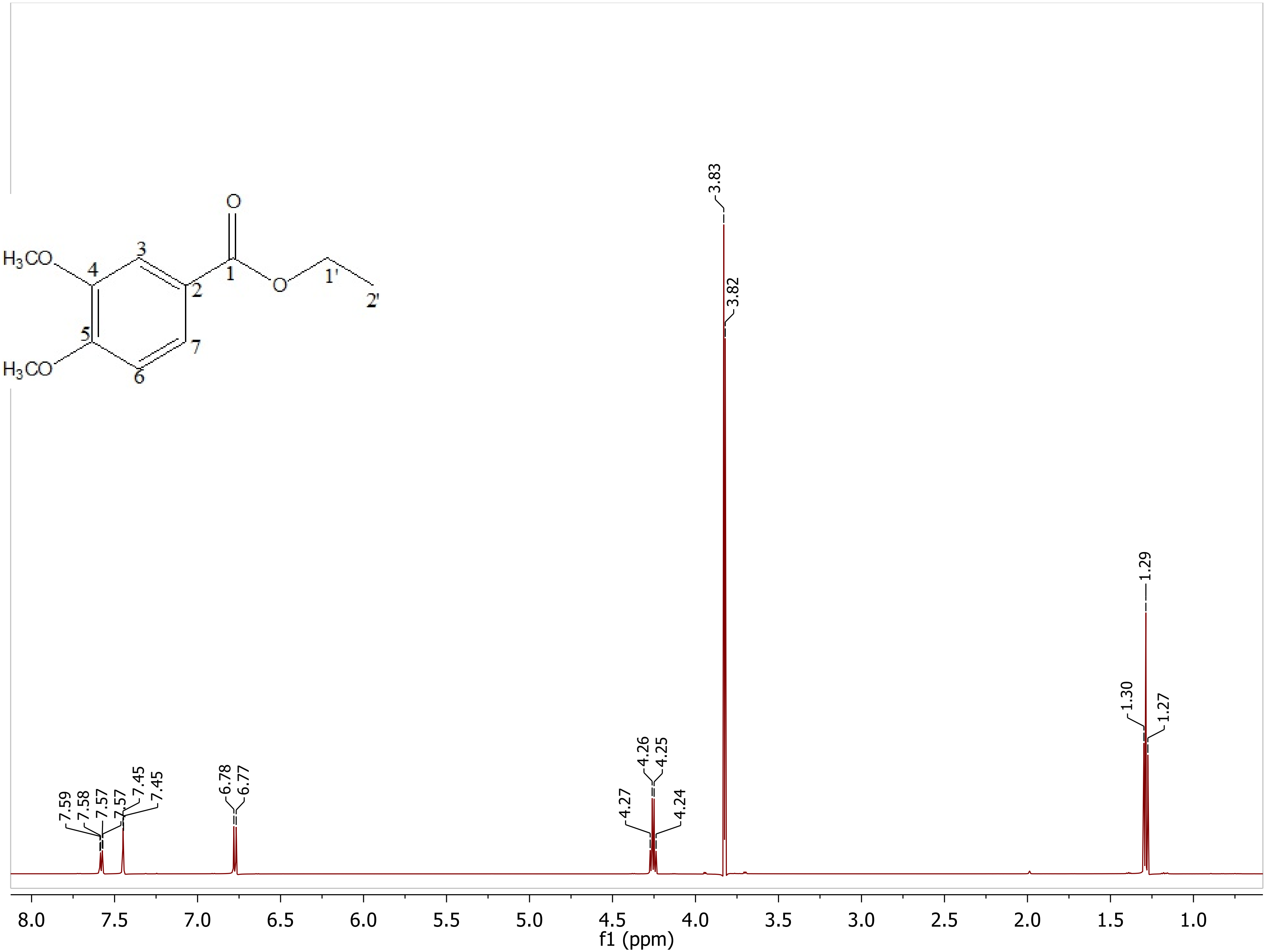

Figure 3S. ^{1}H-NMR ($CDCl_3$, 400 MHz) of compound (**VE**, **1**)

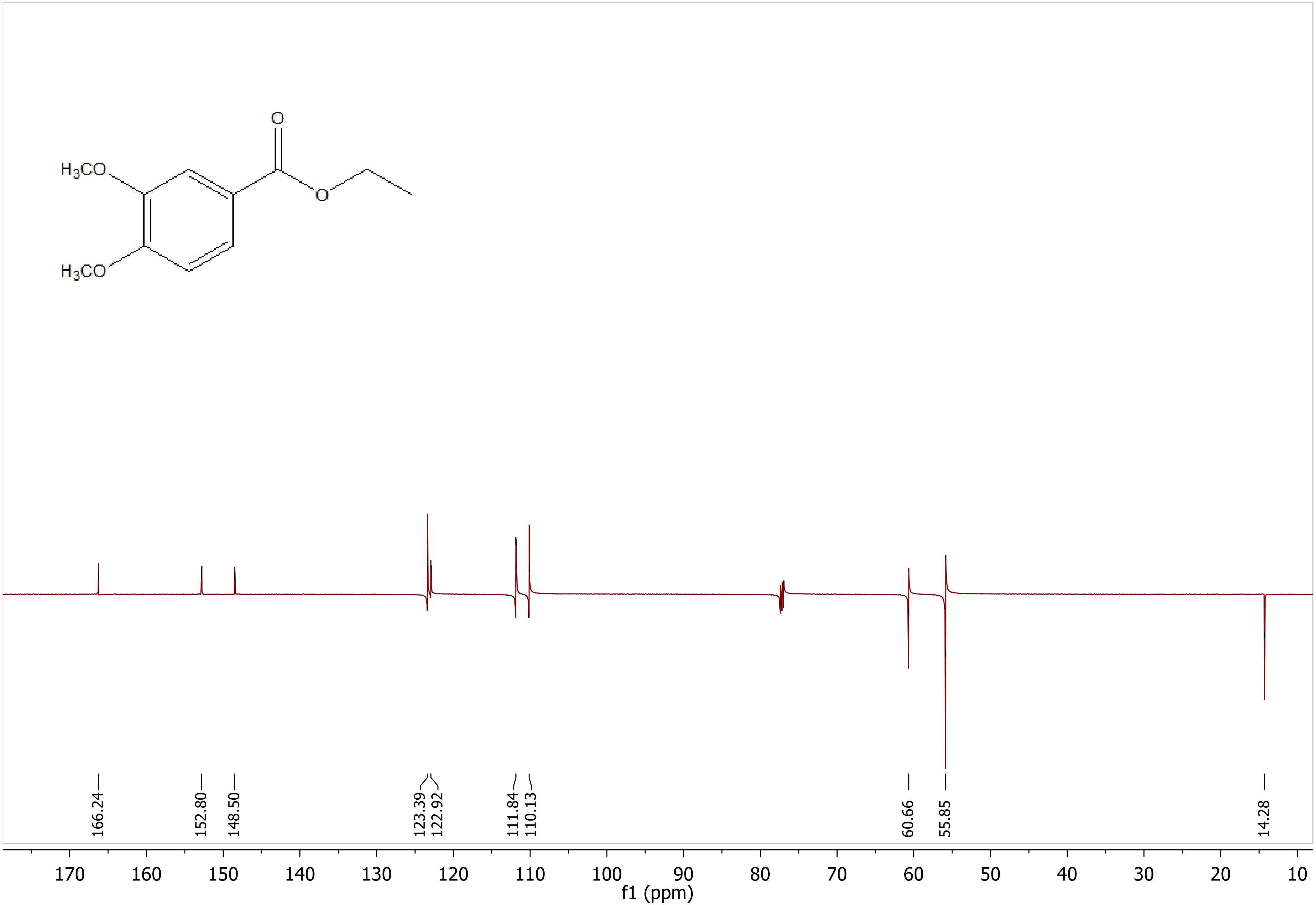

Figure 4S. ^{13}C-DEPT NMR ($CDCl_3$, 100 MHz) of compound (**VE**, **1**)

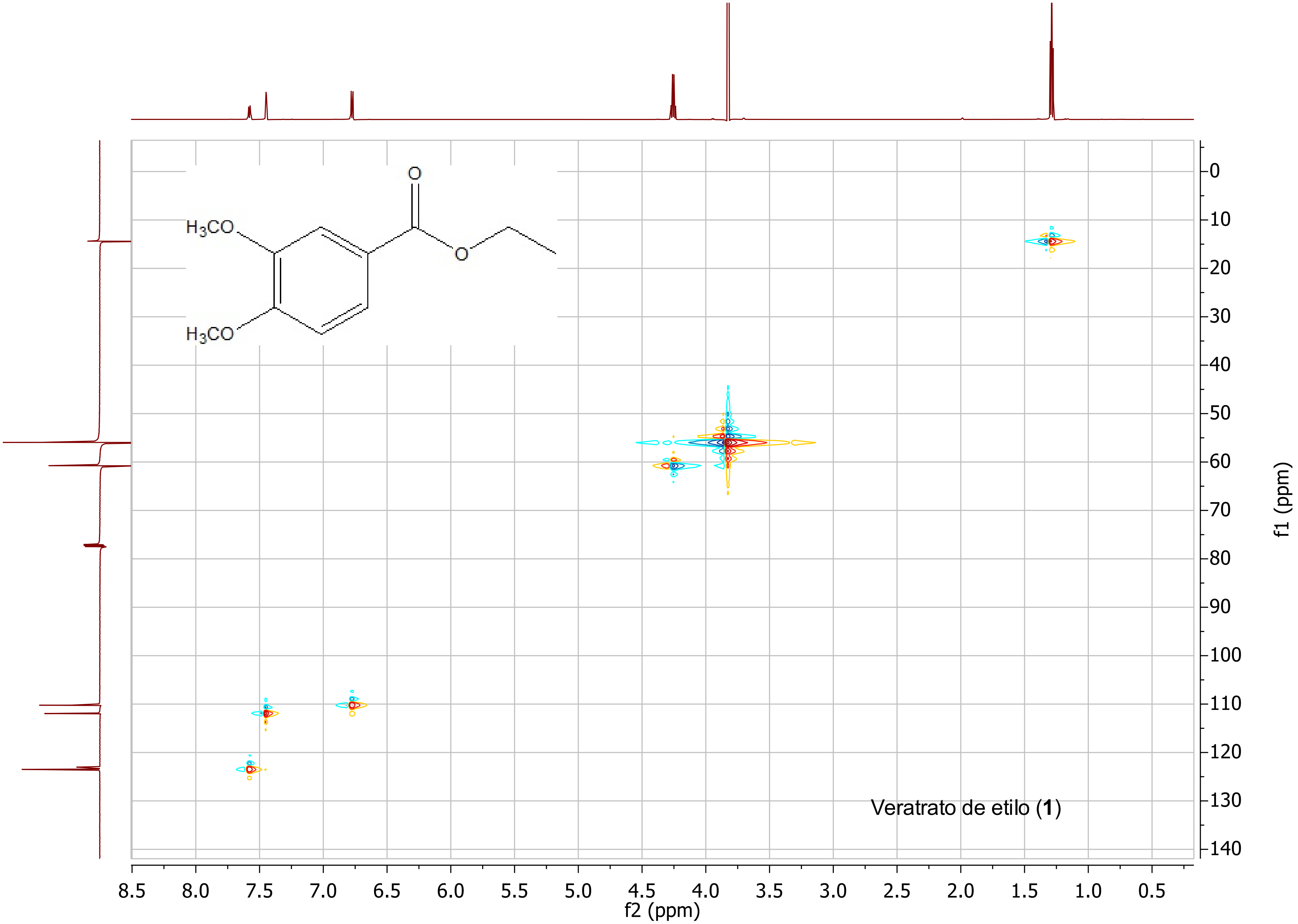

Figure 5S. ^{1}H-^{13}C-NMR HSQC ($CDCl_3$, 400 MHz) of compound (**VE**, **1**)

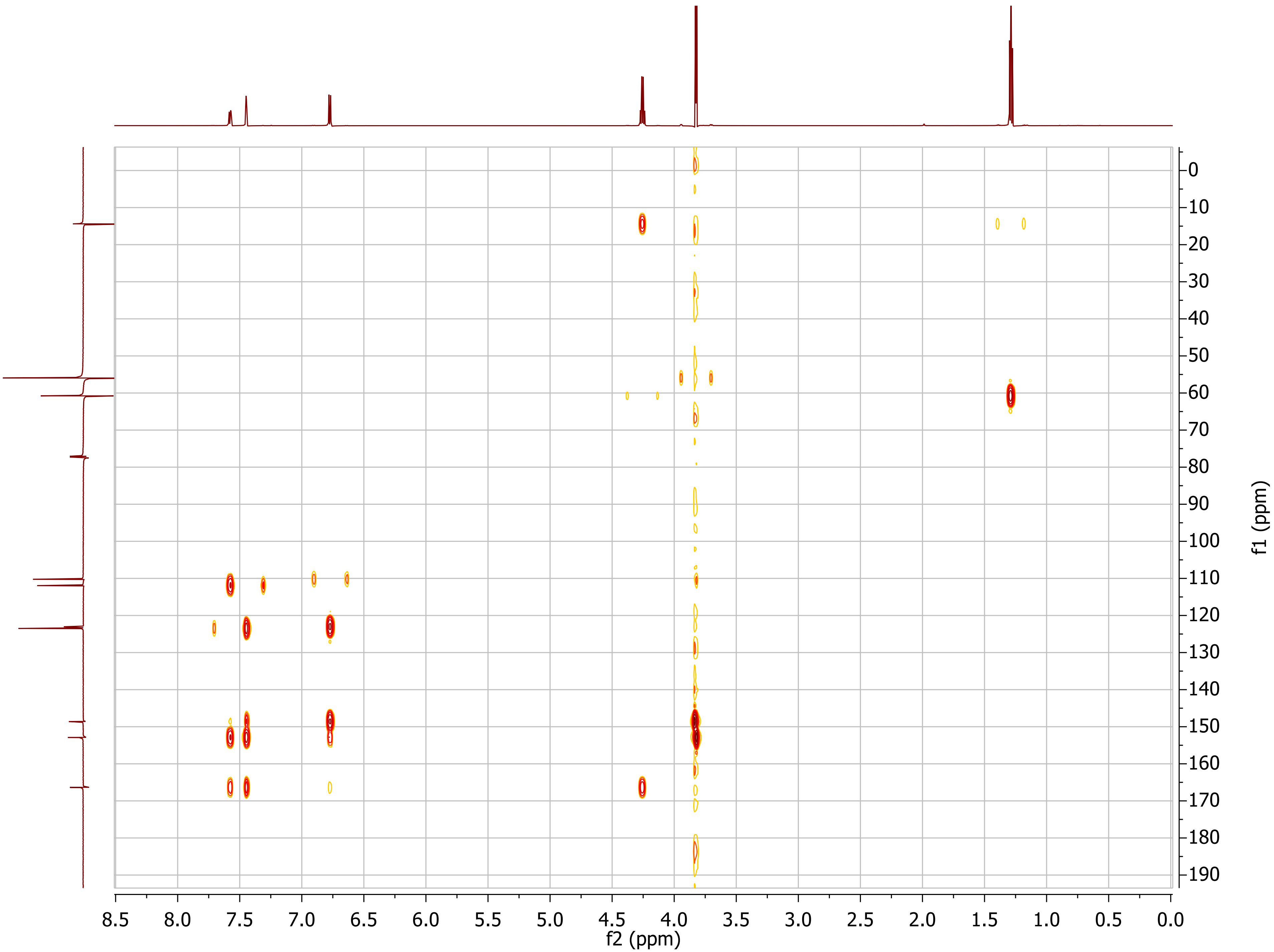

Figure 6S. ^{1}H-^{13}C-NMR HSQC ($CDCl_3$, 400 MHz) of compound (**VE**, **1**)
